# The Effects of Physical Training are Varied and Occur in an Exercise Type-Dependent Manner in Elderly Men

**DOI:** 10.14336/AD.2017.0209

**Published:** 2017-12-01

**Authors:** Mari L. Sbardelotto, Giulia S. Pedroso, Fernanda T. Pereira, Helen R. Soratto, Stella MS. Brescianini, Pauline S. Effting, Anand Thirupathi, Renata T. Nesi, Paulo CL. Silveira, Ricardo A. Pinho

**Affiliations:** Laboratory of Exercise Biochemistry and Physiology, Graduate Program in Health Sciences, Health Sciences Unit, Universidade do Extremo Sul Catarinense, Criciúma, Santa Catarina, Brazil; Laboratory of Exercise Biochemistry and Physiology, Graduate Program in Health Sciences, Health Sciences Unit, Universidade do Extremo Sul Catarinense, Criciúma, Santa Catarina, Brazil; Laboratory of Exercise Biochemistry and Physiology, Graduate Program in Health Sciences, Health Sciences Unit, Universidade do Extremo Sul Catarinense, Criciúma, Santa Catarina, Brazil; Laboratory of Exercise Biochemistry and Physiology, Graduate Program in Health Sciences, Health Sciences Unit, Universidade do Extremo Sul Catarinense, Criciúma, Santa Catarina, Brazil; Laboratory of Exercise Biochemistry and Physiology, Graduate Program in Health Sciences, Health Sciences Unit, Universidade do Extremo Sul Catarinense, Criciúma, Santa Catarina, Brazil; Laboratory of Exercise Biochemistry and Physiology, Graduate Program in Health Sciences, Health Sciences Unit, Universidade do Extremo Sul Catarinense, Criciúma, Santa Catarina, Brazil; Laboratory of Exercise Biochemistry and Physiology, Graduate Program in Health Sciences, Health Sciences Unit, Universidade do Extremo Sul Catarinense, Criciúma, Santa Catarina, Brazil; Laboratory of Exercise Biochemistry and Physiology, Graduate Program in Health Sciences, Health Sciences Unit, Universidade do Extremo Sul Catarinense, Criciúma, Santa Catarina, Brazil; Laboratory of Exercise Biochemistry and Physiology, Graduate Program in Health Sciences, Health Sciences Unit, Universidade do Extremo Sul Catarinense, Criciúma, Santa Catarina, Brazil; Laboratory of Exercise Biochemistry and Physiology, Graduate Program in Health Sciences, Health Sciences Unit, Universidade do Extremo Sul Catarinense, Criciúma, Santa Catarina, Brazil

**Keywords:** aging, strength training, aerobic training, combined training, oxidative stress, inflammation

## Abstract

Regular exercise can decrease the deleterious effects of aging and limit the development and progression of chronic disease in elderly people, depending on the type, intensity, frequency, and duration of exercise. This study aimed to investigate the potential protective effects of different physical training programs on oxidative stress parameters and inflammatory and neurotrophic mediators in the serum of elderly men. Healthy male volunteers [60 to 80 years; n=55] were divided into four groups: control [Ctr, n=14], aerobic training on dry land [ATdl, n=12]; and combined training on dry land [CTdl, n=12] or in water [CTw, n=17]. The training protocols were performed over 8 weeks, three times per week. Each 1 h session included 5 min warming-up exercise, 50 min specific training [aerobic, strength, or combined], and 5 min stretching. Blood samples were drawn 72 h before [baseline] the beginning of the 8 weeks’ protocol and 48 h after the last training session, processed, and the serum was aliquoted and stored at -70 °C until biochemical assessment of oxidative damage, antioxidant system and neurotrophic, growth and inflammatory factors. Elevated BDNF or IGF-1 levels were observed in the ATdl or CTdl groups, respectively. Overall oxidative stress parameters were improved including reduced lipid oxidative damage and increased thioredoxin reductase and glutathione peroxidase activities and total glutathione. Significant decreases in the inflammatory mediators IL-6 and IL-8 were observed; IL-6 was more susceptible to the effects of type of physical training. Thus, the effects of training in elderly men vary in an exercise type-dependent manner.

Human aging is a multifactorial process comprising different variables such as genetics, social conditions and lifestyle [1, 2]. The loss of functional capacity is an inevitable process during this period of life that is especially dependent on the adopted lifestyle [3]. In this sense, physical inactivity is a risk factor that contributes to cardiovascular disease, type II diabetes mellitus, breast cancer and bowel cancer and thus, about 9 % of overall rate of premature death in the western countries [4]. There is a strong evidence that regular exercise can decrease the deleterious effects of the aging process and limit the development and progression of chronic disease in elderly people [1].

Many studies have suggested that aging is directly related to a reduced antioxidant capacity and increased levels of reactive oxygen and/or nitrogen species [5-7]. In any type of tissues, the increased level of free radicles promotes damage in several cellular components and triggers the activation of specific signaling pathways. These effects have been shown to influence numerous cellular processes associated with aging such as sarcopenia and the development of age-related diseases [8].

The skeletal muscle mass decreases about 25% of total bodyweight by age of 75-80 years [9] and muscle strength is reduced 15% from the age of 50, potentially reaching up to 30% [10]. The progressive decline in skeletal muscle mass usually accompanied by decreased muscle strength and functionality which occurs through the process of sarcopenia affecting neuromuscular transmission, muscle architecture, fibers composition, excitation-contraction coupling, and metabolism [11,12]. In additional, studies have also found that elderly people exhibit a chronic low grade inflammatory status associated with elevated serum levels of pro-inflammatory cytokines (TNF-α and IL-6) [13, 14]. This inflammatory condition might cause and/or might be a consequence of cellular oxidative stressmediated by reactive oxygen species (ROS) and/or nitrogen (ERN) and is associated with damage to the skeletal muscles [15, 16].

Other factors including the reduction of serum neurotrophic and growth factor levels also appear to play a crucial role in the health of elderly people. For example, the reduction of brain-derived neurotrophic factor (BDNF) serum levels has been identified as a memory and cognitive deficit as well as a predictor of the risk of morbidity and mortality in the elderly [17] whereas insulin-like growth type 1 (IGF-1) and vascular endothelial growth factor (VEGF) exhibit direct correlation to the reduction of muscle mass and strength in this population [18,19].

Considering the aging population of the developed countries, understanding of the aging has clinical and economical significance. An effective therapy to delay the physiological aging process will have a great impact on population. As oxidative stress plays an important role in these processes, the adoption of strategies that reduce oxidative stress or enhance antioxidant defense systems might yield anti-aging effects. In this context, several studies have shown that regular exercise promotes changes in oxidative stress parameters [20], increases the levels of neurotrophins such as BDNF [21, 22], and modulates growth factors [23] in the elderly. Furthermore, interventions including exercise [both aerobic and resistance training] demonstrate beneficial effects on low-grade chronic inflammatory profiles [14]. However, the type, intensity, frequency, and duration of exercise seem to be decisive for these biochemical changes. In this sense, the aim of this study was to investigate the effects of different physical training programs on oxidative stress parameters and inflammatory and neurotrophic mediators in the serum of elderly men.

**Table 1:** Basal characteristics.

Variable	Control		Training					
			AT		CTdl		CTw	
	Mean ± SD	Min/Max	Mean±SD	Min/Max	Mean ± SD	Min/Max	Mean ± SD	Min/Max
Age (yr)	67.5 ± 6.52	60-78	67.17 ± 4.41	60-75	67.42 ± 6.73	60-78	68.06 ± 6.35	60-79
Body fat (%)	21.41 ± 2.62	16.29-26.55	23.78 ±1.51	20.82-26.75	20.56 ± 2.00	16.63-24.48	24.86 ± 1.18	22.55-27.17
Lean mass (kg)	57.01 ± 1.88	53.32-60.69	65.35 ± 2.00	61.43-69.27	56.91 ± 1.73	53.51-60.31	61.90 ± 1.55	58.87-64.94
BMI (Kg.m^-2^)	25.18 ± 1.72	21.80-28.57	28.93 ± 1.04	26.90-30.97	24.48 ± 0.75	23.02-25.95	27.75 ± 0.91	25.96-29.53

## MATERIAL AND METHODS

### Trial Design and participants

It is a parallel randomized controlled trial conducted at a physical fitness complex in the state of Rio Grande do Sul, Brazil. The recruitment of the participants was conducted through the radio and the randomization was conducted by lot. The eligibility criteria to participate in this study was: no use of drugs or medications capable of inducing myopathy, no physical limitations or clinical diseases that might compromise the execution of the exercise, and at least 6 months immediately prior to the study without regular exercise. All selected subjects were instructed not to perform any type of physical training out of the study.

### Randomization and blinding

**Figure 1.**
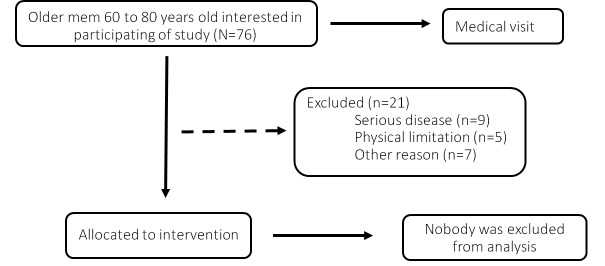
The flowchart of the participants.

A total of seventy six elderly people were interested to participate in this study but 21 elderly did not meet the inclusion criteria. The flowchart of the participants is described in figure 1. After the initial screening to observe the number of participants in fulfilling the inclusion criteria, the researchers wrote numbers 1-4, indicating each group, in small a piece of papers, folded It and put in an opaque plastic bag. In exceptional cases, some participants were allocated in the groups to medical advice regardless of draw. Fifty five participants were randomly divided into four groups: control (Ctr, n = 14), aerobic training on dry land (ATdl, n = 12); combined training on dry land (CTdl, n = 12); and combined training in water (CTw, n = 17). The sample size was not calculated. We chose the approach to widely publicize the research in a local radio and conducted the research with as many participants. The participants received explanations regarding the purpose and risks of the protocol and signed to accept the consent term. Participant characteristics are listed in Table 1. The study was approved by the Research Ethics Committee of the Universidade do Extremo Sul Catarinense (protocol number 119477/72012).

**Table 2:** Aerobic training program.

Mesocycle	Microcycle (Weeks)	Sessions per mesocycle	Series per session	Time of exercise in the series	Intensity (% Fcmáx)	Time of active recovery between series	Total time
1	1 - 2	6	5	5 min	70%	1 min	29 min
2	3 - 5	9	4	8 min	75%	1 min	35 min
3	6 - 8	9	3	15 min	80%	2 min	47 min

### Training protocols

Training protocols were performed over a total period of 8 weeks, three times per week. Each 1 h session was divided into three parts: 5 min warming-up exercise, 50 min specific training (aerobic, strength, or combined, see Tables 2, 3, and 4, respectively), and 5 min stretching exercise.

### Blood sample

Blood samples (10 mL) were drawn 72 h before [baseline] the beginning of the 8 weeks’ protocol and 48 h after the last training session. The blood samples were drawn from the antecubital vein, collected in vacutainers without additives, and centrifuged at 1500 rpm for 10 min at 4°C. Aliquots of washed/lysed red blood cells and serum samples were stored at -70°C until biochemical assays were performed.

**Table 3:** Combined training program on dry land.

					Strength exercise			Aerobic exercise		

Mesocycle	Microcycle (Weeks)	Sessions per mesocycle	Series per session	Number of repetition in the series	Intensity % 1RM	Time of active recovery between series	Time of exercise in the series	Intensity (% Fcmáx)	Time of active recovery between series	Total time
1	1 - 2	6	3	12 X	60%	2 min	5 min	70%	1 min	36 min
2	3 - 5	9	3	10 X	70%	2 min	5 min	75%	1 min	45 min
3	6 - 8	9	3	08 X	80%	3 min	5 min	80%	1 min	68 min

### Oxidative damage assessment

The malondialdehyde (MDA) concentrations in the serum samples were determined by reverse phase high performance liquid chromatography (Agilent Technologies 1200 series; Santa Clara, CA, USA) according to Grotto et al. [24] using a thiobarbituric acid derivatization. A standard curve was prepared using malondialdehyde tetrabutylammonium salt at concentrations ranging from 0.5 to 5.0 µM. Results were expressed as µmol/L MDA. Protein carbonylation was determined according to the method described by Levine *et al*. [25]. Protein carbonyl content was measured by labeled protein-hydrazone derivatives using 2,4-dinitrophenylhydrazide. The concentration of 2.4-dinitrophenylhydrazide was measured as absorbance difference against 2.4-dinitrophenylhydrazide blank at 370 nm. The Sulphydryl groups content was determined using the 5-5°- dithiobis (2-nitrobenzoic acid) method (DTNB) (Sigma, St. Louis, MO, USA). The reaction was initiated by adding 30 µL DTNB (10 mM) to phosphate-buffered saline. After 30 min of incubation at room temperature, the absorbance at 412 nm was measured and the amounts of 5,5°-Dithiobis [2-nitrobenzoic acid] formed (equivalent to the amount of sulphydryl groups) was calculated using the technique developed by Aksenov and Markesbery [26].

### Antioxidant system assessment

Catalase (CAT) activity was determined based on the hydrogen peroxide (H_2_O_2_) decomposition rate generated by the enzyme present in the sample using a 10 mM H_2_O_2_ solution in potassium phosphate buffer, pH 7.0 [27]. The maximum H2O2 decomposition rate was measured at 240 nm; the values were expressed as CAT units per mg protein. The glutathione peroxidase (GPx) activity was determined according Flohé and Gunzler [28]. An aliquot of the serum sample and 10 mL tert-butylhydroperoxide (tBuOOH) were added to a mixture formed by a reaction medium. The tBuOOH catalyzed glutathione oxidation [GSH to GSSG] by GPx activity, which leads to the oxidation of NADPH as measured in a plate reader at 340 nm. The values were expressed as mM NADPH/min/mg protein. Thioredoxin reductase (TrxR) activity was measured as the NADPH dependent colorimetric reduction of DTNB [29]. The samples were homogenized in 1M potassium phosphate buffer and enzyme activity was determined by DTNB reduction by NADPH with absorbance measured at 412 nm for 1 min. TrxR activity was expressed in Units/mL/mg protein. Total GSH levels were determined as described by Hissin and Hilf [30] with modifications. GSH was measured in serum after protein precipitation with 10% trichloroacetic acid. An aliquot of the sample was added to phosphate buffer with 500 μM DTNB. Color development resulting from the reaction between DTNB and the thiols reached a maximum in 5 min and was stable for more than 30 min. Absorbance was read at 412 nm after 10 min. A standard curve of reduced glutathione was used to calculate the GSH levels in the samples.

### Neurotrophic, growth factor, and inflammatory parameter assessment

The serum levels of BDNF, IGF-1, interleukin 1β (IL-1β), interleukin 6 (IL-6), interleukin 8 (IL-8), and interleukin 10 (IL-10) were determined by commercially available ELISA kits (R&D Systems, Minneapolis, MN, USA).

### Protein assay

Protein levels were measured in all samples using the Bradford method [31], which is based on an absorbance (595 nm) shift of the dye Coomassie brilliant blue G-250 in which the red form of the dye is converted into its bluer form upon binding to the protein in the sample. Protein standards were obtained by diluting a stock solution of bovine serum albumin. Linear regression was used to determine the actual protein concentration of each sample.

**Table 4:** Combined training program in water

*Mesocycle*	Microcycle (Weeks)	Sessions per mesocycle	Series per session	*Strength exercise*			*Aerobic exercise*			Total time
				Runtime in the series	Speed of execution	Time of active recovery between series	Time of exercise in the series	Intensity (% Fcmáx)	Time of active recovery between series	
*1*	1 - 2	6	2	30 sec	maximum	1 min	10 min	70%	1 min	44 min
*2*	3 - 5	9	3	20 sec	maximum	1 min	15 min	75%	1 min	49 min
*3*	6 - 8	9	4	15 sec	maximum	1 min	20 min	80%	1 min	59 min

### Statistical analysis

The data were expressed as the means ± standard error of the mean [SEM] and represent the number of times that the trained groups changed their values compared to control and baseline groups. The Kolmogorov-Smirnov test was used to confirm normal distribution of the values of all analyzed parameters. The Student’s t-test was used to compare baseline versus post-training measures and one-way analysis of variance (ANOVA) followed by a Bonferroni *post hoc* test was used to detect the statistical difference between trained groups versus the control group. Differences were considered to be significant if *P* < 0.05. SPSS (version 21.0; Chicago, IL, USA) was used for all statistical analyses.

### Limitations

This study has some limitations due to specificity of the population. We were not able to control the use of medications fully and stratify shorter age groups. Although, we know that Dual-energy X-ray absorptiometry (DXA) is a reference assessment technique in muscle mass evaluation and it has been considered as a gold-standard technique for sarcopenia diagnosis. Unfortunately, we did not measure the body composition by this method due to technical limitations at the time of data collection.

## RESULTS

**Figure 2.**
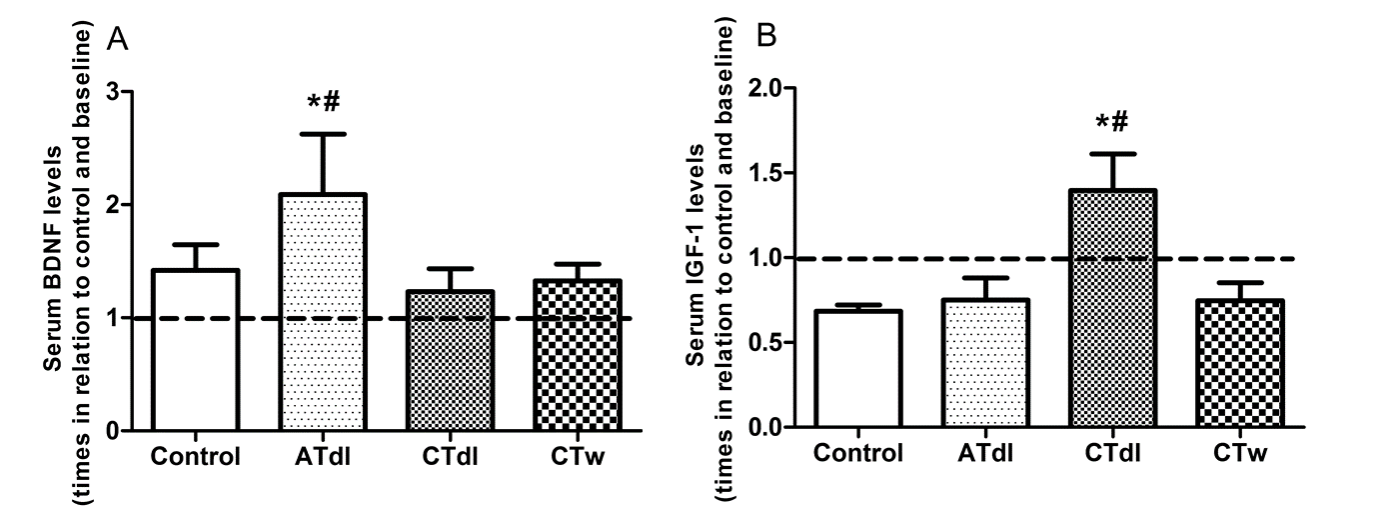
Effect of different physical training protocols on BDNF (A) and IGF-1 (B) levels 55 elderly men performed over a total period of 8 weeks’ sessions of aerobic training on dry land (ATdl), combined training on dry land (CTdl), or combined training in water (CTw). The dotted line represents the baseline. The values are presented as the means ± standard error of the mean (SEM) and the results represent the number of times compared to baseline* or to the control group# considering a significance index of P > 0.05 according to one-way ANOVA followed by Bonferroni’s post-hoc test.

Neurotrophic and growth factor changes in the elderly occurred in an exercise type-dependent manner: The levels of BDNF and IGF-1 were measured as neurotrophin and growth factor markers respectively. The results showed increased levels of BDNF and IGF-1 after physical training compared to baseline and control group levels (Fig. 2); however, these changes occurred in an exercise type-dependent manner. The BDNF levels were elevated in the ATdl group (Fig. 2A) whereas IGF-1 exhibited enhanced levels in the CTdl group (Fig. 2B).

**Figure 3.**
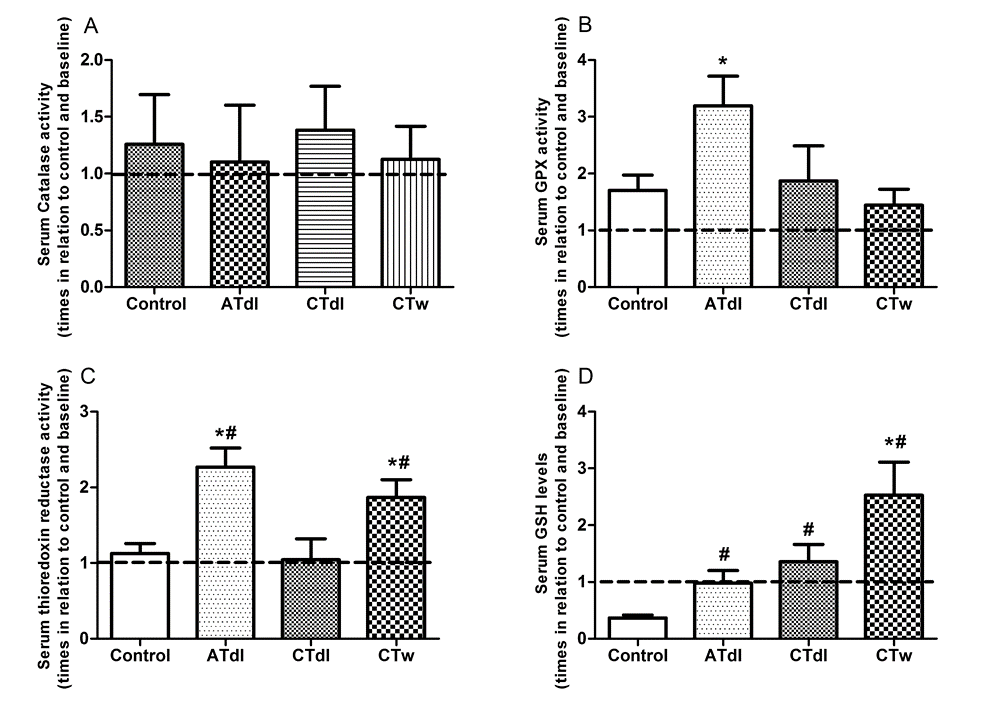
Effect of different training protocols on antioxidant system in elderly men after two months of physical exercise aerobic training on dry land (ATdl), combined training on dry land (CTdl), or combined training in water (CTw) were performed three times a week for 60 min per session over a total period of 8 weeks. The dotted line represents the baseline. The values of catalase (**A**), GPx (**B**) and TRxR (**C**) activities and GSH levels (**D**) are presented as the means ± standard error of the mean (SEM) and the results represent the number of times compared to baseline* or to the control group# considering a significance index of *P* > 0.05 according to one-way ANOVA followed by a Bonferroni’s post-hoc test.

Effect of different physical training protocols on age-induced changes in antioxidant levels in the serum: The antioxidant system was evaluated from measures of CAT, GPx, and TrxR activities along with total GSH/GSSH in the sera of elderly men (Fig. 3). No significant difference was found in the enzymatic activity of CAT (Fig. 3A). In contrast, GPx and TrxR activities were significantly changed. The ATdl group showed increased activity of GPx compared to baseline (Fig. 3B) whereas the CTw group showed enhanced activity of TrxR in relation to baseline as well as the control group (Fig. 3C) in both the ATdl and CTw groups. GSH, the major low-molecular-weight antioxidant in serum, showed enhanced levels in all trained groups compared to the control group; however, only the CTw group showed elevated levels compared to baseline (Fig. 3D).

**Figure 4.**
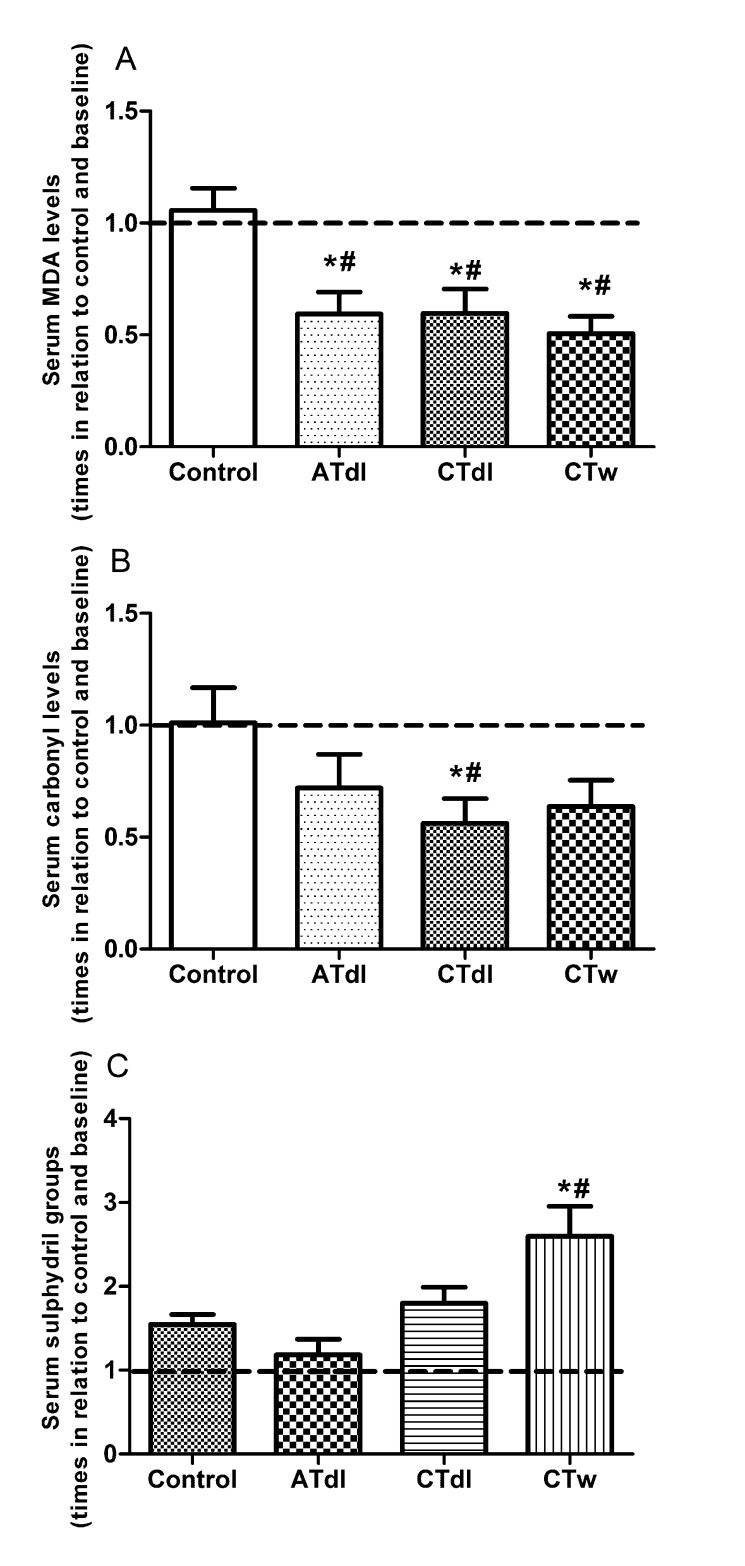
Changes in MDA (A), carbonyl groups (B), and Sulphydryl (C) levels induced by different training protocols in the elderly men Two months of physical training with aerobic training on dry land (ATdl), combined training on dry land (CTdl), or combined training in water (CTw) were performed three times a week for 60 min per session over a total period of 8 weeks. The dotted line represents the baseline. The values are presented as the means ± standard error of the mean (SEM) and the results represent the number of times compared to baseline* or to the control group# considering a significance index of P > 0.05 according to one-way ANOVA followed by a Bonferroni’s post-hoc test.

Increased oxidative damage in elderly men could be reversed by physical training: In this study, we investigated lipid damage (MDA levels) and the oxidation and modification of proteins via carbonyl group formation and the oxidation of sulphydryl groups (Fig. 4). Reduced levels of MDA were observed in all groups after physical training (Fig. 4A). Similarly, all groups showed reduced levels of carbonylated protein, but only the CTdl group exhibited values that were significantly decreased compared to both baseline and the control group (Fig. 4B). In contrast, sulphydryl group oxidation was not observed in the ATdl and CTdl groups whereas the CTw group showed enhanced levels of sulphydryl groups compared to baseline and the control group (Fig. 4C).

**Figure 5.**
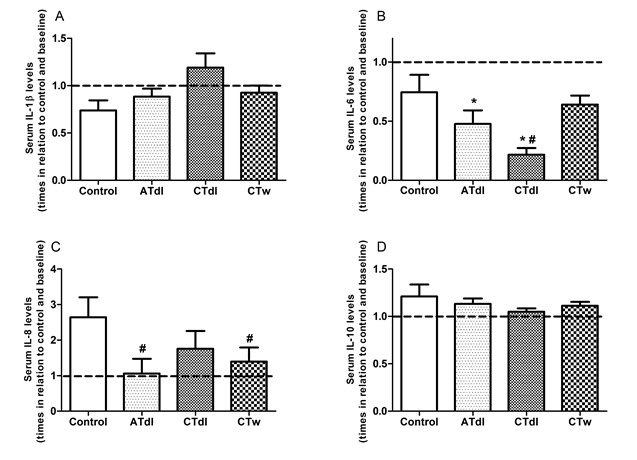
Effect of different training protocols on anti and pro-inflammatory parameters in elderly men. Two months of physical training with aerobic training on dry land (ATdl), combined training on dry land (CTdl), or combined training in water (CTw) were performed three times a week for 60 min per session over a total period of 8 weeks. The dotted line represents the baseline. The values of IL-1β (A), IL-6 (B), IL-8 (C) and IL-10 (D) levels are presented as the means ± standard error of the mean (SEM) and the results represent the number of times compared to baseline* or to the control group# considering a significance index of P > 0.05 according to one-way ANOVA followed by a Bonferroni’s post-hoc test.

Physical activity modified the inflammatory profile in an exercise type-dependent manner in elderly men: Anti- and pro-inflammatory interleukins were used to determine the inflammatory profile of elderly men (Fig. 5). The levels of IL-1β (Fig. 5A) and IL-10 (Fig. 5D) in the serum of the trained groups were unaltered compared to baseline and the control group. In contrast, the ATdl group showed significantly decreased levels of IL-6 (Fig. 5B) compared to baseline and of IL-8 (Fig. 5C) compared to the control group, whereas the CTdl group exhibited reduced IL-6 levels in relation to baseline and the control group. CTw treatment significantly reduced the levels of IL-8 only in relation to the control group.

## DISCUSSION

Muscle wasting and loss of functional ability are frequently observed in the elderly and previous studies have suggested that these alterations might be partially associated with the constant and cumulative effects of ROS [32, 33]. Accordingly, physical exercise has been considered as one of the possible strategies to regulate the cellular redox state of elderly subjects [7, 34] as well as to reduce age-related muscle decline [2]. Although physical exercise has been proven to limit the development and progression of chronic disease and reduce the deleterious effects of aging, the mechanisms underlying the favorable effects of exercise during this phase of life are not fully understood.

Changes in the levels of neurotrophic and growth factors have been observed during aging that have been associated with sarcopenia [35]. For example, the anabolic effect of the IGF-1 growth factor on muscle tissue has been shown to stimulate muscle cell proliferation and differentiation, facilitate muscle protein synthesis, and inhibit its degradation [36]. Previous studies have also shown that low serum levels of IGF-1 were associated with losses in muscular strength, cognitive function, physical performance, and mortality in the aging population [37-39]. BDNF is a neurotrophin related to growth, differentiation, and neuronal plasticity that has many peripheral sources such as epithelial and vascular cells [40], macrophages and leucocytes [41], and muscle cells [42], which all have been shown to synthesize and release BDNF. Notably, reduced BDNF serum concentrations were consistently observed in adulthood psychiatric pathologies [43], and both IGF-1 and BDNF levels have been associated with the development of sarcopenia [35, 44].

Our results showed increased levels of BDNF and IGF-1 after physical training compared to baseline and the control group. Although it has been proposed that IGF-1 might interface with BDNF-mediated synaptic plasticity in the brain during exercise [38], this interface does not seem to manifest at the systemic level because the changes observed in our study occurred in an exercise type-dependent manner. BDNF levels were elevated in the ATdl group whereas IGF-1 levels were increased in the CTdl group. Differences observed in both the ATdl and CTdl groups might be related to the type of training, as these two types of exercise differ primarily by the intensity of muscular contractions involved as well as the means by which energy is generated within the muscle. Combined exercise involves numerous muscle groups and mainly improves muscle resistance and strength while aerobic training has the central focus on improving cardiorespiratory fitness. Previous studies have shown that both BDNF and IGF-1 are dependent on the type of exercise performed. For example, Forti and colloegues [45] recently showed that only a mixed-low-resistance training program with a very high number of repetitions at a sufficiently high external resistance was able to increase circulating BDNF in older male participants. Furthermore, Goekint et al. [46] showed that strength training in sedentary male subjects did not influence serum BDNF. In addition, Taekema et al. [47] observed a relationship between IGF-1 levels and exercises for muscle strength in the oldest of elderly women.

In addition to training type, two other factors might also be associated with changes in BDNF and IGF-1 levels in the sererum of elderly individuals: 1) the levels of oxidative stress and 2) the ability of physical training to modulate the muscle antioxidant defense system. Oxidative stress might influence numerous cellular processes linked to aging and the development of age-related diseases [8, 48] that are accompanied by muscle wasting [49]. According to Meng and Yu [6], the pathogenesis of sarcopenia is multifactorial and can be attributed to different factors such as oxidative stress, inflammation, endocrine changes, physical inactivity, and under-nutrition. Nonetheless, many of these factors do not act alone and many of their causal pathways intersect or overlap in relation to oxidative stress response. Therefore, a reduction in oxidative damage or increased efficacy of the antioxidant defense systems would likely promote significant effects toward anti-aging. Thus, the reduced levels of oxidative damage observed in our study might imply a reduction in ROS production or an improvement in the antioxidant defense system. Furthermore, this redox balance might be the major factor underlying the increase in BDNF and IGF-1 levels.

The enzymatic antioxidant system is the first line of cellular defense against ROS generation. Age-related changes in the expression and activity of antioxidant enzymes have been described in different tissues. In our study, the ATdl group showed increased activity of GPx compared to baseline alone whereas the CTw group showed enhanced activity of TrxR in relation to baseline as well as the control group in both the ATdl and CTw groups. Both GPx and TrxR-dependent peroxiredoxin proteins remove hydrogen peroxide and improve the control of intracellular redox states. From the results obtained using an experimental model, Sullivan-Gunn and Lewandowski [50] suggested that the decline in antioxidant protection by catalase and GPx is indicative of antioxidant dysfunction and might therefore act as a major contributing factor in the development or onset of sarcopenia. Nevertheless, there is no plausible explanation for the difference in the activity of these proteins between the different training models. However, aerobic training has been suggested as an important model to improve the enzymatic antioxidant defense system in humans [51, 52]. In addition, we observed elevated levels of GSH in all trained groups compared to the control group and following CTw compared to baseline. GSH plays a central role in antioxidant defenses and evidence from several human studies suggests that the concentrations of GSH decline with aging [53-55]. In response to exercise, the GSH levels at rest tend to increase as a cellular adaptation to the production of ROS-induced aging. In our study, CTw showed a more pronounced training effect on GSH levels. This suggests that CTw might exert a different stimulus on the glutathione system in the aged. In addition, the stimuli of strength and muscle strength associated with the aerobic components might have made a difference in the results obtained. Additional research is necessary to investigate this question further.

The positive effects of physical training on the antioxidant system reduce the possibility of oxidative damage in biomolecules. Among the oxidative damage markers analyzed in this study, the MDA level exhibited the most significant effect from physical training. Reduced levels of lipidperoxidation were observed in all groups after physical training as well as in relation to the control group. The low presence of end-products of lipid peroxidation in the serum reflects conditions of reduced levels of oxidative stress. Likewise, [56] recently showed decreased lipid peroxidation after 16 weeks of combined physical training in healthy middle-aged men and in elderly individuals. This result suggests that the effect of training on lipidperoxidation occurs in an exercise type-independent manner in the elderly. In contrast, the antioxidant system changes observed in the current study suggested that the antioxidant system is dependent on the type of exercise performed. Therefore, the antioxidant defense system in serum might contain other antioxidant defense mechanisms that promote cellular protection and an equal response from different types of training. On the other hand, De Conzalo-Calvo and collegues [34] showed increased lipid peroxidation in middle-age men and in an aged population following long-term training with combined endurance and resistance activities. In this case, the time factor might have influence the elevated level of lipid peroxidation observed. Taken together, these data suggest that both the time and the type of exercise practiced by the elderly are crucial in the lipid peroxidation reduction induced by exercise.

Oxidative stress and inflammation are dependent processes that together are involved in many diseases associated with aging [14]. Age-related intracellular redox unbalance appears to be a primary causal factor in producing a chronic state of low-grade inflammation [6]. Notably, aging results in slight elevations of circulating proinflammatory mediators [57] and in alterations in T-cell function, immune cell senescence, alteration of the extracellular matrix, and increased fat mass and foci of chronic infection [14]. However, previous studies have also shown that regular physical exercise might mediate the anti-inflammatory response [14, 58, 59]. Thompson et al. [59] showed that the levels of IL-6 in serum decreased after 12 weeks of moderate resistance training in healthy middle-aged men compared to sedentary controls. Our results showed that the levels of IL-1β and IL-10, representing pro- and anti-inflammatory cytokines in the serum of the trained groups were not altered. Conversely, in the ATdl group the IL-6 levels were decreased significantly compared to baseline and those of IL-8 were reduced compared to the control group whereas the CTdl group exhibited reduced IL-6 levels in relation to both baseline and the control group. In comparison, the CTw group showed significantly reduced levels of IL-8 only in relation to the control group. Previous studies have shown that elevated serum IL-6 in aging is associated with different markers of physical ability. Taaffe and coauthors [60] found that IL-6 was positively linked with low walking speed and poor muscle strength and Cesari et al. [61] showed that elevated IL-6 levels were significantly associated with poor physical performance and muscle strength in older individuals. Therefore, although the overall interleukin levels are altered by exercise, IL-6 appear to be more susceptible to the effects of physical training than IL-8 because the trained groups had lower levels of IL-8 in relation to the control group but these levels were not significantly reduced after the training programs.

In conclusion, the mechanisms of systemic adaptation induced by different models of training on dry land and in water are distinct, wherein each type of exercise activates different neurotrophic and growth factors as well as variably affects the regulation of cellular oxidative stress and inflammatory parameters. Taken together, these results show that the systemic changes related to aging mediated by physical training are dependent on the type of training accomplished; *i.e.,* the effects of training are varied and occur in an exercise type-dependent manner. This study has important clinical implications because it suggests that the type of exercise practiced in the aged is directly related to modification of the desired parameters, which contradicts the standard dogma that the adherence to exercise is more important than the type of exercise performed.
